# Endoscopic removal of a large ingested squeeze ball at the duodenojejunal flexure using multiple devices

**DOI:** 10.1055/a-2058-8879

**Published:** 2023-04-26

**Authors:** Yoshimasa Kubota, Tesshin Ban, Takuya Takahama, Shun Sasoh, Tsuyoshi Itoh, Yoshinori Nakamura, Takashi Joh

**Affiliations:** 1 Department of Gastroenterology and Hepatology, Gamagori City Hospital, Gamagori, Aichi, Japan; 2 Department of Gastrointestinal Surgery, Gamagori City Hospital, Gamagori, Aichi, Japan

Clinical guidelines recommend endoscopic removal using a basket catheter for blunt and spherical foreign bodies in the small bowel; however, objects with a diameter more than 2.0–2.5 cm cannot typically be passed through the pylorus 1 . This article describes the endoscopic removal of a large ingested squeeze ball at the duodenojejunal flexure using multiple devices.

An 80-year-old man with dementia, who was in a rehabilitation facility following cerebral infarction, was referred to our department because of severe vomiting. Computed tomography revealed a radiolucent, capsulized, and spherical object of 44 mm in diameter at the duodenojejunal flexure. After multidisciplinary discussion, endoscopic removal was planned using a colonoscope (PCF-H290TI; Olympus, Tokyo, Japan).

Following endoscopic recognition of the object, a large balloon catheter (Huge; XEMEX, Tokyo, Japan) was advanced with the aim of withdrawing the object to the duodenal bulb to avoid its migration beyond the duodenojejunal flexure ( Fig. 1 and Fig. 2 a ; Video 1 ); however, a basket lithotriptor (LithoCrush V; Olympus) could not be deployed because the lumen space was occupied. Therefore, alligator forceps (FG-49L-1; Olympus) were used to grasp the object, which appeared to be made of a stretchable, yet unbreakable, material ( Fig. 2 b ). After the object had been withdrawn over the pyloric ring, an obstruction was encountered as it passed through the esophagogastric junction. We then used a basket lithotriptor to grasp the object instead and it was identified as a squeeze ball ( Fig. 2 c ). Finally, the squeeze ball was extracted perorally, without complications.

**Fig. 1:**
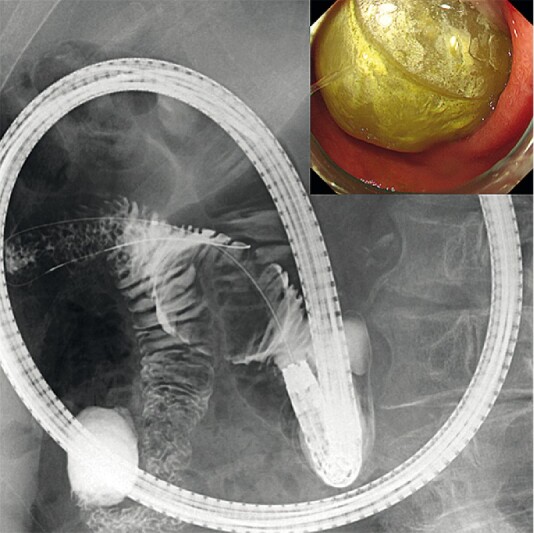
Fluoroscopic image showing a radiolucent spherical object at the duodenojejunal flexure, which could have migrated toward the anal side owing to peristalsis. An endoscopic view (inset) showed a yellow-colored artificial object.

**Fig. 2:**
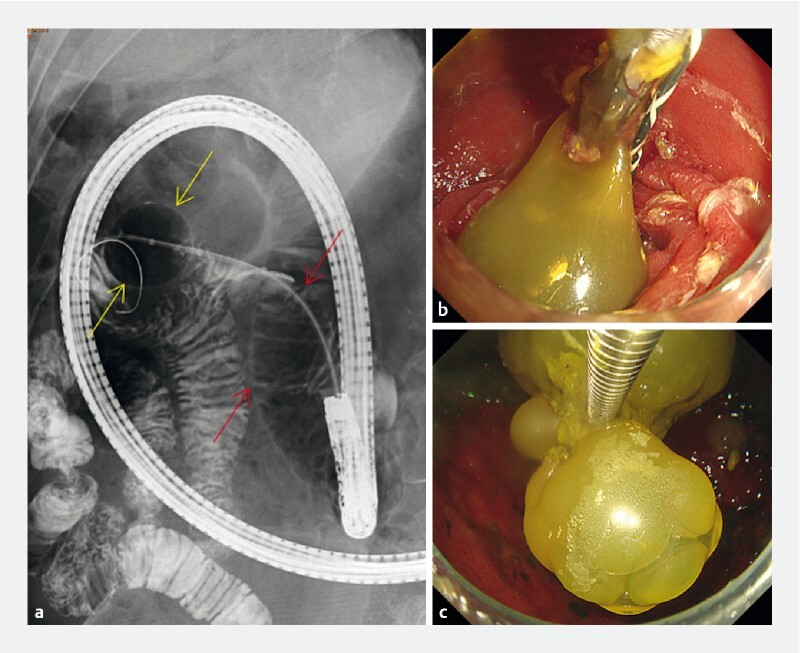
Images during the endoscopic removal of an ingested object using multiple devices showing: **a** fluoroscopic view of a large balloon catheter (yellow arrows) advanced to the anal side of the object (red arrows) and being used to withdraw the object to the duodenal bulb; **b** endoscopic view of alligator forceps being used to grasp the object, which was revealed to be made of a stretchable, yet unbreakable, material; **c** a basket lithotriptor being used to grasp the object, which revealed it to be a “squeeze ball,” prior to its quick extraction via the mouth.

**Video 1** Endoscopic removal of a large ingested squeeze ball, initially found at the duodenojejunal flexure, using multiple devices.


Squeeze balls, which mimic jelly candies, are used in rehabilitation facilities. If ingested by a disoriented person, these deformable objects may pass through natural constrictions, including the piriform recess, esophagogastric junction, and pyloric ring. The high deformability of the squeeze ball enables it to pass through natural constrictions under traction force. Devices with a high gripping ability are helpful for endoscopic removal.

Endoscopy_UCTN_Code_TTT_1AQ_2AH

## Notes

Endoscopy E-Videos

https://eref.thieme.de/e-videos
*E-Videos* is an open access online section of the journal *Endoscopy* , reporting on interesting cases and new techniques in gastroenterological endoscopy. All papers include a high-quality video and are published with a Creative Commons CC-BY license. Endoscopy E-Videos qualify for HINARI discounts and waivers and eligibility is automatically checked during the submission process. We grant 100% waivers to articles whose corresponding authors are based in Group A countries and 50% waivers to those who are based in Group B countries as classified by Research4Life (see: https://www.research4life.org/access/eligibility/ ). This section has its own submission website at https://mc.manuscriptcentral.com/e-videos
